# Medical students with performance difficulties need wide support: Initial results of an academic tutoring program

**DOI:** 10.6061/clinics/2021/e2495

**Published:** 2021-03-15

**Authors:** Patrícia Lacerda Bellodi, Marisa Dolhnikoff, Academic Tutoring Group-FMUSP, Alfredo Luiz Jacomo, Alexander Augusto de Lima Jorge, Alexandre Archanjo Ferraro, Ana Claudia Camargo Gonçalves Germani, Ana Cristina Aoun Tannuri, Beatriz Helena Carvalho Tess, Bruno Caramelli, Denise Maria Avancini Costa Malheiros, Iolanda de Fátima Lopes Calvo Tibério, José Pinhata Otoch, Luiz Fernando Ferraz da Silva, Luiz Henrique Martins Castro, Maria Cláudia Nogueira Zerbini, Marisa Dolhnikoff, Milton de Arruda Martins, Rogério de Souza, Rossana Pulcineli Vieira Francisco

**Affiliations:** Departamento de Cirurgia; Departamento de Clínica Médica; Departamento de Pediatria; Departamento de Medicina Preventiva; Departamento de Cirurgia; Departamento de Medicina Preventiva; Departamento de Cardiopneumologia; Departamento de Patologia; Departamento de Clínica Médica; Departamento de Cirurgia; Departamento de Patologia; Departmento de Neurologia; Departamento de Patologia; Departamento de Patologia; Departamento de Clínica Médica; Departamento de Cardiopneumologia; Departamento de Obstetrícia e Ginecologia; I Nucleo de Apoio ao Estudante (NAE), Faculdade de Medicina FMUSP, Universidade de Sao Paulo, Sao Paulo, SP, BR.; II Centro de Desenvolvimento de Educacao Medica (CEDEM), Faculdade de Medicina FMUSP, Universidade de Sao Paulo, Sao Paulo, SP, BR.; III Departamento de Patologia, Faculdade de Medicina FMUSP, Universidade de Sao Paulo, Sao Paulo, SP, BR.

**Keywords:** Tutoring, Medical Students, Remediation, Academic Performance

## Abstract

**BACKGROUND:**

Even students with previous academic success may face challenges that affect their academic performance. Many medical schools offer programs to students at the risk of academic failure, to ensure that they succeed in the course.

**OBJECTIVE AND METHODS:**

In this report we describe a pioneering academic tutoring program developed at a Brazilian medical school and discuss the initial results of the program based on the feedback from tutors and data regarding the progression of students in the medical course.

**RESULTS:**

In 2018, 33 students enrolled into the program. Students' performance difficulties were mainly associated with mental health problems and socioeconomic vulnerability. Of the 33 students, 27 (81.8%) were assisted by the Mental Health Support Service and 16 (48.5%) were assisted by the Social Assistance Service. In addition to the planning academic activity class load, tutors were able to assist students in solving socioeconomic issues, carrying out personal support interventions with the promotion of self-esteem, and presenting suggestions for behavioral changes in their routine. For most students (72%), the action plan proposed by the tutors was successful. Eight of the 14 (57%) students in the fourth year progressed to the final two years of in-hospital practical training (internship).

**CONCLUSIONS:**

The Academic Tutoring Program showed positive results for most of the students. Close monitoring and tutor intervention allowed students with poor academic performance to overcome the low performance cycle. These important tasks demand time and energy from tutors, and institutional recognition of these professionals is essential for the successful maintenance of the program.

## INTRODUCTION

In the long and demanding journey of medical education, even students with a previous history of academic success may face challenges that can affect their performance and eventually lead them to situations of academic vulnerability, preventing them from advancing in the course. Only a small proportion of medical students are faced with medical course insufficiency. Nevertheless, poor performance in the course is of utmost concern to medical educators, which impacts the student body, professors, and their programs (1). Early identification of difficulties in acquiring medical knowledge and skills is crucial to ensure safe medical practice. The fact that medical students with difficulties also become doctors with difficulties emphasizes the importance of early identification and intervention of low performance students (2,3).

Medical students with academic difficulties are a heterogeneous group. It is widely recognized that the reasons underlying poor performance are varied. The causes for academic failure include both academic and non-academic factors, such as ineffective learning and study skills, lack of resilience and inadequate stress management, difficulty in managing study load, incompatibility with the learning environment, socioeconomic difficulties, and mental health (3,4).

In Medical Education, interventions with underperforming students involve “remediation”, “the act of facilitating a correction for trainees who started out on the journey toward becoming a physician, but have moved off course” (1). As a developmental education concept, remediation incorporates comprehensive efforts to help students mature both academically and personally throughout the course (5). In addition to identifying the most appropriate study methods, supporting students in academic vulnerability involves understanding the determinants of academic performance, including motivational and environmental factors (6).

Based on the difficulties involved and the teaching context in which the process takes place, remediation can assume different forms. In general, the process begins by identifying the needs to be addressed and the objectives to be achieved. Following this, the action plans of teaching and/or additional training are developed. Subsequently, there is monitoring, and, lastly, a new evaluation to verify if the objectives were achieved (1). The development of an individualized action plan is one of the key elements of an effective remediation program, in which the mentor/tutor plays a pivotal role (6).

Previous reports indicate the importance of these interventions, which are needed to be carried out in the first years to interrupt the students’ low performance cycle, who, without guidance, tend to repeat failures. However, the remediation programs have focused more on improving the students’ performance on a given assessment or exam, than on helping them develop effective lifelong learning skills. To date, there is little evidence available to guide the best remediation practices for different levels of medical training. Previous studies indicate that there is a need for remediation processes that focus more on “learning to learn” and less on “exam coaching.” In addition, the studies show that the remediation processes must take into account not only the nature of the students' problems, but also the availability and interest of teachers, who often resist participation due to additional work load or doubts regarding the effectiveness of the process (3,6).

Many medical schools believe that appropriate and timely support can help students with academic performance difficulties overcome experiences of failure, and have offered programs to students at the risk of failure to thrive on the course. Interventions not only tend to reinforce knowledge or skills, but also include additional strategies such as small tutoring groups, laboratory review, peer-facilitated learning, and academic mentoring.

In this report, we describe the implementation of an academic tutoring program developed in a Brazilian medical school and present the initial results of the program based on the feedback of tutors and data regarding the progression of students in the medical course.

## METHODS

### FMUSP Academic Tutoring Program

In 2017, the “Faculdade de Medicina da Universidade de São Paulo” identified a significant number of students in a situation of academic vulnerability. This led to them creating a task force to evaluate this situation and propose measures to improve the network support of these students. Students in academic vulnerability were identified by the repeated failures in individual courses, falling behind by one or more years in the course.

At that time, the medical school was undergoing a curricular-restructuring process, and students with previous failures needed adaptations in order to transition to a new academic scenario.

After a few months, an Academic Tutoring Program was structured with the aim of creating a support strategy for students. Carried out by professors, the program was dedicated toward the individual tutoring of students with low academic performance. The program was not a remediation program in the classic sense, that is, a new opportunity to learn content or practices. The primary objectives of the program were to help students identify and understand their difficulties, help them manage and organize their academic schedule, and refer students to other support programs when deemed necessary.

The Academic Tutoring Program was structured in the context of a novel organization, the Student Support Center (NAE), that provided pre-existing psychopedagogical, psychological, and social services. Additionally, the medical school offers a Mentoring Program to all students, dedicated toward the personal and professional development of students through the exchange of experiences between the faculty members and students organized in small groups.

The following parameters were defined as the central aspects of the Tutoring Program:

- **Model:** We adopted the model of “tutoring per academic year,” ascribing three to four tutors per academic year (1^st^ to 6^th^ year of medical school), who were all faculty members involved in different courses. The recruited faculty were responsible for the students at risk for that specific year.
- **Tutors’ profile and role:** The tutors must be faculty members with interest and who are available to participate in the program. These tutors were invited by the program coordinator. Twenty-one teachers were invited to make up the initial group of tutors. Tutors would have to assess the situation of students, understand the reasons for academic difficulties, prepare a tutorial action plan with students, facilitate students’ communication with the coordinators of courses, and refer the students to complementary support programs when deemed necessary (such as a mental health support program).
- **Students’ role**: Students must attend meetings with their respective tutors and/or the program coordinator, participate in the preparation of the tutorial action plan, and provide regular feedbacks to their tutors regarding the progress of their actions.
- **Flow:** Students could access the program through spontaneous demand, by an active search by the program coordination, or by a referral from other faculty members or peers.
- **Active search:** Students who had failed three or more courses and students facing difficulties to finish pre-requisites to initiate the practical in-hospital training (internship) due to curricular transition were invited to participate in the program by the program coordinator via a private email message.
- **Program coordination**: It required one faculty member (MD) and one professional with experience in Mentoring Programs (PLB). The coordination was responsible for inviting and training academic tutors, structuring the program, actively searching for the students at risk, pairing students and tutors, and monitoring and evaluating the program through academic records and periodic meetings with the tutors.

### Data collection

The program’s success was evaluated based on two parameters: a) tutor feedback after a one-year interval, and b) data regarding the progression of students in the medical course post a two-year follow-up.

We also obtained data on the number of students assisted by other support services (Mental Health Support Service and Social Assistance Service).

The tutors were asked to respond to an online questionnaire that addressed the following points:

- Initial situation of the student (failures and difficulties),
- Proposed action plan,
- Current situation (at the end of the evaluation period),
- Number of approvals in the previously failed courses,
- (If a fourth-year student) The ability of the student to complete all the pre-requisites to be admitted during the last two years of in-hospital medical training (internship),
- Number of meetings held in the period, and
- The positives, negatives, and the suggestions made by the tutors

### Data analysis

Data are presented descriptively and qualitatively. The report excerpts are presented for illustration purposes.

## RESULTS

The Academic Tutoring Program was approved in December 2017 and the activities started in January 2018. Of the 21 faculty members who were sent an invitation by the program coordinator, 16 faculty members agreed to participate, one did not respond, and four declined the invitation for lack of available time. The final tutor group included 17 faculty members from 12 departments (including the program coordinator who was also a tutor). They were involved during all the six years of the medical course. For the active search, we sent emails to 55 students, including 30 students with three or more course failures and 25 students in curriculum transition.

In 2018, 33 students participated in the tutoring program (from a total of 1,050 medical students); 21 were men (63%) and 12 were women (37%). There was a predominance of 4^th^ (14 students) and 3^rd^ year (11) students, followed by 1^st^ year (4 students), 2^nd^ year (2 students), and 5^th^ and 6^th^ year students (one student each).

Most of the students searched the program based on spontaneous demand (19 students, 57.5%). Only seven students joined the program in response to the program coordination’s active search. As attendance is optional, students who did not respond to the active search are reevaluated the next year and invited again if they happen to remain in academic vulnerability. The other seven students were referred in a varied manner (four students by the Academic Service, two students by the Student Support Center/NAE, and one student by a faculty member).

Ninety academic tutoring meetings were held by the tutors, with approximately three to four meetings with a single student per year. In addition to in person meetings, several tutors also reported contacts and guidance via email and Whatsapp messages. Three students did not respond to the tutors’ initial contact.

In addition to academic tutoring, a section of the students were assisted by other support programs: 27 (81.8%) by the Mental Health Support Service, 16 (48.5%) by the Social Assistance Service, and one by the Psychopedagogical Support.

We have not yet evaluated the students who entered the program in 2019. For those who were in the program in 2018, the two-year follow-up showed that the proposed action plan was successful for 24 students (72%). These students could move ahead in the course, including 8 of the 14 4^th^ grade students (57%) who participated in the in-hospital training internship. Several failures in individual courses could be resolved, which ranged from one to seven per student.

### Feedback from tutors

The students faced academic difficulties for the following reasons: Students' performance problems were mainly related to mental health factors, socioeconomic issues, or both. Learning difficulties or lack of interest among students were not important issues; they were rarely related to low performance.

1. Mental health: Depression, its impact on the ability to concentrate, organize, and motivate, was prevalent. Anxiety symptoms and conditions such as panic syndrome, phobia, and eating disorders were also reported.
2. Socioeconomic vulnerability: Socioeconomic difficulties, such as remote place of residence with a long daily commute, also contributed to the performance difficulties faced by some students. Students received one or more types of academic scholarships and/or economic aid for housing, food, and book purchase.
3. Other issues such as health problems, difficulties in managing schedules, and difficulties in communicating with the administrative staff and course coordinators were also associated with failures.

### Role and actions of tutors

The role of academic tutors is complex and comprehensive, involving different types of interventions. Academic tutors provide practical academic guidance, mediation with teachers of specific courses, emotional support, encouragement to seek help in mental health, and help to address the socioeconomic needs. In most cases, these interventions were performed simultaneously, comprising of different aspects of the needs of students.

### Program achievements and failures

The following statements present examples of the satisfaction received by tutors while solving some issues:

“The meeting was very positive. The student's performance was surprising and exceeded my expectations. He/she is enrolled in all courses this semester, which are running normally. The student is doing well.”

“So far, he/she has passed in the courses. He/she continues to use the medication and is concerned about some unforeseen event or any new failure, but he/she seems excited, is happy with results so far, and has been attending classes.”

Reasons for failure included the students not attending the first meeting, limited time available for a large number of issues to be resolved, and absenteeism from the process, often resulting from the worsening of students' mental health. Nine students did not have any significant improvement during the period; six of them had many failures in individual courses when they entered the program, which certainly contributed to their failure. Of the nine students, three entered the program too late, two had significant fluctuations in their mental health, two did not attend most meetings, and two did not respond to the tutor's initial contact. The following are some of the statements made by tutors regarding the students’ failure:

“I don't know if I was able to help him/her because he/she missed two other meetings due to other appointments. He/she seemed very dependent on social approval”.

“Difficult situation, as he/she may not be able to pass this year's courses. From the point of view of mental health, he/she seems controlled. There was an initial good response, with greater involvement in studying and adherence to tutoring. But maybe too much time has passed.”

The main positive points highlighted by the tutors are bonding between the students and tutors, students’ perception that the tutoring program is beneficial, and students' gratitude for overcoming the difficulties. The main negative points highlighted by the tutors are student absences, late search for help, difficulties in contacting the teaching staff, and the institutional insufficiencies in the student support system.

## DISCUSSION

Our initial experience with the Academic Tutoring Program revealed a series of interesting and important elements that will help us understand the potential and the challenges to the support intervention program meant for medical students with academic difficulties.

It is well known that most medical students do not need any kind of remediation as they progress through the course. Those who do, however, require a disproportionate amount of staff and administrative time and resources (1). Our results are in line with those findings.

Academic tutors, although initially needed for solving specific academic issues, assisted students in a much more comprehensive way, playing different functions and roles. In addition to providing support for the preparation of an action plan, tutors were able to assist students in overcoming socioeconomic issues (by referring them to social service and guiding them to apply for stipends), they carried out personal support interventions with self-esteem promotion, and presented suggestions for behavioral changes in students’ routine, with particular attention to mental health preservation.

This comprehensive support, both personal and academic, seems to be essential for the remediation of students with performance difficulties. It has been recognized that this type of support requires experienced faculty members who are capable of playing a unique combination of roles (that of a facilitator, a nurturing mentor, a disciplinarian, a diagnostician, and a role model) with high levels of teaching presence and practical wisdom (7). An effective remediation process not only involves the identification of the goals and objectives to be achieved, but also the development of a relationship with the student, where a non-judgmental attitude enables a decline in resistance and increases the odds of the process being considered as an opportunity for growth (8).

Careful listening of our students not only led the tutors toward a greater and better understanding of the students' difficulties, but also toward an understanding of the institutional difficulties in attending to their different needs. In many cases, implementation of tutor-proposed strategies meant that the student received psychosocial assistance.

It is a known fact that depression and burnout are highly prevalent in medical students, along with them having a lower quality of life than the other students of the same age group (9,10). A recent review and meta-analysis reported a high proportion of Brazilian medical students to be suffering from various mental health problems, including anxiety, depression, common mental disorders, and alcohol abuse, with a combined prevalence of 30 to 33%. An approximate 13% students experience burnout and half of them experience poor sleep quality (11). In order to carry out the proposed action plan, it is necessary that a student is “well enough” with regard to his/her mental health. The well-being and mental health status of learners, especially in the context of anxiety and depression, have been increasingly recognized as powerful influences while engaging with learning and retaining knowledge. Often, poor academic performance is just the “tip of the iceberg”, and the identification of the underlying psychosocial issues is very important in order to understand and address the problem (5). In this sense, the institutional limitations to offer psychological assistance was considered by the tutors as a hindering factor that made it difficult for many students to achieve faster and more positive outcomes.

While the prevalence of depression and common mental disorders are higher among female students, they were however, more prevalent among the male students of our program. This might possibly be due to the predominance of male students in our medical school. Previous studies have shown that while mood and anxiety disorders are more frequent among female students, burnout prevails more among male students (12).

The students also faced socioeconomic difficulties that were associated with mental health problems. This association is observed in Brazilian studies carried out in both public and private institutions, showing that economic factors are associated with low quality of life and that mental health can be affected when everyday tensions are related to socioeconomic status (13).

Studies show that students who struggle to find housing, those who cannot find an appropriate place to study, and those who do not have the support of others tend to perform poorly in exams. A safe environment, physically and emotionally, is critical to have a good academic performance (14). Therefore, institutional insufficiencies of social support resources (stipends, housing, and others) were also pointed out by the tutors as a limiting factor in the academic assistance offered to students with performance difficulties.

It is important to emphasize that greater access to higher education, with an increase in both the number and diversity of students, has raised important concerns about student permanence. Equality and diversity in admission have enabled the entry of students from different cultural, social, and ethnic backgrounds to medical schools; as well as the entry of students with special, physical, and psychological needs (5). When this change in profile is combined with the rigorous and specific demands of academic work, the risk of failure is greater, underscoring the need to provide support to the students at risk; thus, resulting in the increase in studies on the effectiveness of remediation (3).

Another important role played by academic tutors was to facilitate communication between students and course coordinators. In addition to the already known stressors, such as pressure for high performance, a recent study brings out the importance of organizational aspects, such as information systems with inadequate flow, little flexibility, and difficulty in contacting the faculty and the administrative sector as important stress generators for students (15).

One of the most challenging aspects of remediation is the early identification of students at risk of failure to help them thrive in the course. It has come to be known that early interventions have a greater potential to interrupt the low performance cycle. However, many of the remediation efforts are carried out during the last years of medical school. Considering that these students have little belief in their self-efficacy and have negative feelings about learning, experiencing success as soon as possible can help them develop a greater sense of control over their learning and performance (3). It has come to be known that students with difficulties are reluctant to be identified. A non-punitive, reliable, and transparent remediation process in a confidential and nurturing environment is the key for remediation success (6,16).

Another important issue, reported in previous studies and also present in our experience, concerns the great demand of time and emotional involvement of tutors. The time spent by tutors not only includes periodic meetings with students to develop specific academic plans, but also mediation with the course coordinators, interaction with psychological and social support groups, and regular meetings with other tutors and program coordinators to exchange experiences. Using too much of their time to help a relatively small number of students with performance difficulties is a common complaint among the teachers involved in remediation (1). Additionally, it is well known that the medical school faculty tends to hold pessimistic views in the academic recovery or professionalism of the students at risk. It remains unclear as to who should confront this issue, as well as how to improve knowledge and skills for effective remediation strategies (8). Tutors are often faced with complex situations, which lead them to reflect on the limits of their role and how to deal with situations that are “irremediable.” The anxieties derived from these situations are of concern to worldwide educators because of the lack of satisfactory alternatives available in the medical profession to those who are unable to complete their training (1).

Recognition of the tutor's work is another point raised by the faculty involved in remediation. Despite the recognition of the importance of remediation, the tutor's work is little valued by their peers. Objective ways of valuing these professionals must be considered to ensure that the lack of recognition does not function as a disincentive for the staff involved in this program.

Our program has not been evaluated by the students yet. However, up until now, their informal feedback, whether to academic tutors or to the coordinator, has been very positive. Not only is the program perceived as a space of concrete and effective help, but the tutors have been recognized as a reliable source of support with a non-judgmental attitude. This not only helps the students from a practical standpoint, but also motivates them to move forward in the course.

The following are some of the examples of students’ feedback:

“Teacher, good afternoon! This is Z, the student you helped to plan the semester. I got all my grades and would like to thank you very much for your help. In the end, everything worked out! Shall we plan another meeting at the beginning of classes? Thank you very much for your attention.”

“I am totally grateful to the tutoring program; it was the first great support I received from the university after a long process of illness.”

## CONCLUSIONS

Providing support to students, who do not reach the expected performance standards at some point, has been considered as a part of the responsibility of medical schools. We believe that our Academic Tutoring Program can fulfill this role. Positive feedback, both from students and academic tutors, despite the difficulties pointed out, brings out the importance of continued effort. Close monitoring and tutor interventions allowed to interrupt the students’ low performance cycle. This important task demands time and energy from tutors. Institutional recognition of these professionals is essential for the successful maintenance of the program.

Our initial experience shows that the Academic Tutoring Program already has an important place within the institutional support actions. It holds an important place for not just being a strategy that helps students with difficulties to express their potential, but also for expanding the view of faculty toward the students’ difficulties in their academic life.

### Academic Tutoring Group - FMUSP (alphabetical order) Faculdade de Medicina FMUSP, Universidade de Sao Paulo, SP, BR

**Alfredo Luiz Jacomo** (Departamento de Cirurgia), **Alexander Augusto de Lima Jorge** (Departamento de Clínica Médica), **Alexandre Archanjo Ferraro** (Departamento de Pediatria), **Ana Claudia Camargo Gonçalves Germani** (Departamento de Medicina Preventiva), **Ana Cristina Aoun Tannuri** (Departamento de Cirurgia), **Beatriz Helena Carvalho Tess** (Departamento de Medicina Preventiva), **Bruno Caramelli** (Departamento de Cardiopneumologia), **Denise Maria Avancini Costa Malheiros** (Departamento de Patologia), **Iolanda de Fátima Lopes Calvo Tibério** (Departamento de Clínica Médica), **José Pinhata Otoch** (Departamento de Cirurgia), **Luiz Fernando Ferraz da Silva** (Departamento de Patologia), **Luiz Henrique Martins Castro** (Departmento de Neurologia), **Maria Cláudia Nogueira Zerbini** (Departamento de Patologia), **Marisa Dolhnikoff** (Departamento de Patologia), **Milton de Arruda Martins** (Departamento de Clínica Médica), **Rogério de Souza** (Departamento de Cardiopneumologia), **Rossana Pulcineli Vieira Francisco** (Departamento de Obstetrícia e Ginecologia).

## AUTHOR CONTRIBUTIONS

All authors contributed substantially to the conception and design of the study, and to the analysis and interpretation of data. Bellodi PL and Dolhnikoff M prepared the original draft of the manuscript. All authors reviewed and approved the final version of the manuscript.
